# Characterized Functional Groups of Temperature- and Salt-Resistant Copolymers and Surfactants and Their Relationships

**DOI:** 10.1021/acsomega.3c04369

**Published:** 2023-08-23

**Authors:** Huiming An, Leilei Zhang, Li Zhao, Qing Guo, Weiwei Nan, Wenwen He

**Affiliations:** Bailie School of Petroleum Engineering, Lanzhou City University, Lanzhou 730070, China

## Abstract

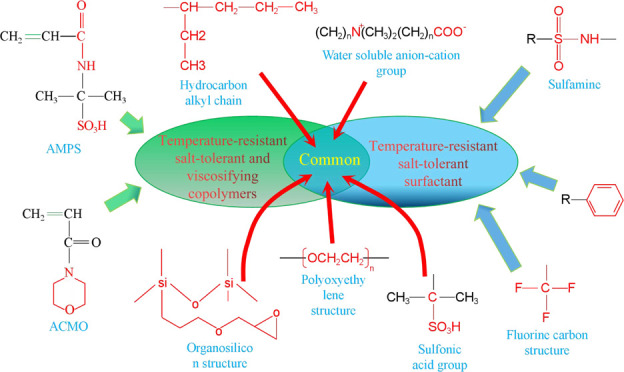

As the physical properties of newly discovered oil and gas reservoirs become more complex and the requirements for field development effectiveness continue to increase, the performance of polymers and surfactants, which are commonly used as chemical agents in oil field development, is facing great challenges. The determinations of temperature and salt resistance of anti-temperature and anti-salt viscosity-enhancing copolymers and low-interfacial-tension surfactants in recent years have been reviewed. The mechanism of temperature and salt resistance of distinct functional groups was discussed, and the common functional groups of temperature- and salt-resistant viscosity-increasing copolymers and temperature- and salt-resistant low-interfacial-tension surfactants were pointed out. An outlook on the molecular structure design of a new temperature- and salt-resistant oil displacement agent is presented.

## 1 Introduction

Polymers and surfactants are widely used in oil and gas field development processes such as drilling, formation modification, and tertiary oil recovery.^1,2^ Drilling fluid dynamic to plastic ratio, lubrication adjustment, fracturing fluid viscosity, and rejection suitability, the viscosity of displacement fluid, and oil–water interfacial tension requirements require a series of chemical agents for adjustment.^3,4^ Existing oil fields are generally facing the actual situation of increasing water content and decreasing production in old oil areas, and the development of untouched, hard-to-reach blocks is gradually on the agenda.^5,6^ In recent years, the adjustment of national policy on petrochemical energy has led to an increase in the number of unconventional oil and gas field development projects.^7,8^ The accompanying physical conditions of oil and gas reservoirs have become more complex and demanding, especially high temperature and high salt, making the performance of commonly used chemical agents in oil fields degrade and fail to meet the target requirements, which becomes a common factor limiting the efficient development of oil and gas fields.^9,10^ Improving the temperature and salt resistance of chemical agents for oil fields has become a hot research direction in oil fields. Many scholars have conducted a lot of research on the viscosity-building and low interfacial tension properties of oil displacement agents for oilfield development under high temperature and high salt, and many new temperature- and salt-resistant copolymers and surfactants have been synthesized and their properties have been measured.

There are numerous factors influencing the viscosity-increasing properties of copolymers and the low interfacial tension properties of surfactants. Many studies start with external conditions, such as temperature, pressure, and mineralization. Studying the effect of external conditions on chemical performance stops at the level of chemical performance evaluation. The composition and structure of the molecules are the fundamental controlling factors for the viscosity-increasing properties of the copolymers and the low interfacial tension properties of the surfactants, and they determine the conditions under which the chemicals are applied. An in-depth study of the molecular composition and structure of temperature- and salt-resistant copolymers and surfactants is an important guarantee for improving the performance of chemical agents and expanding the applicable conditions.

This paper reviews the molecular structures and properties of some typical temperature- and salt-resistant viscosity-enhancing copolymers and low-interfacial-tension surfactants. The mechanisms of viscosity increase and reduction of interfacial tension by the introduced functional groups under high-temperature and high-salt conditions were analyzed in conjunction with physicochemical principles. The relationship between temperature- and salt-resistant viscosity-enhancing copolymers and temperature- and salt-resistant low-interfacial-tension surfactants was found from the perspective of molecular property functional groups. Their commonalities in the molecular structure are pointed out to provide a reference for the design of the molecular structure of the new temperature- and salt-resistant oil displacement agent.

## 2 Results and Discussion

### 2.1 Temperature- and Salt-Resistant Viscosity-Increasing Copolymer

The polymer can effectively reduce the water–oil mobility ratio and increase the displacement fluid sweep volume in tertiary recovery.^3,11^ Partially hydrolyzed polyacrylamide (HPAM) linear polymers break their main chains under high-temperature degradation and mechanical shear, forming oligomers with shorter chain lengths, leading to a decrease in the viscosity of their aqueous solutions. Inorganic salt dehydration and metal ion compressive diffusion bilayer effects cause polymer macromolecules in solution to curl and decrease the hydrodynamic radius, leading to a decrease in viscosity.

#### 2.1.1 Synthesis of Viscosity-Enhancing Copolymers and Their Properties

**Figure 1:**
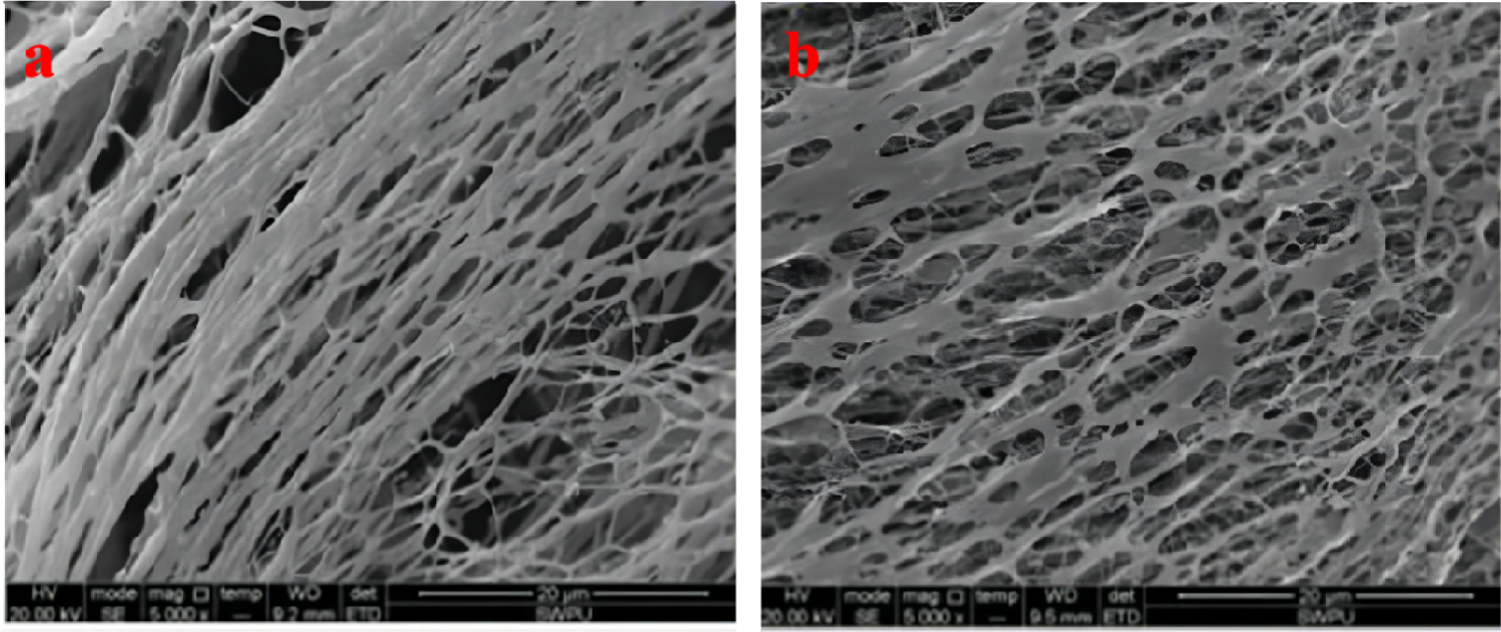
SEM images of HPAM (a) and hyperbranched hydrophobically conjugated polyacrylamide with sulfonic acid groups (b).

Zhao et al.^12^ synthesized a hyperbranched hydrophobically conjugated polyacrylamide containing sulfonic acid groups, as shown in the structural Formula 1. As shown in Figure 1, the hyperbranched copolymer showed an obvious net-like structure in aqueous solution with a mineralization of 10,000 mg/L at 90 °C, while the HPAM without the branching structure showed an obvious linear strip structure. The intermolecular branching structure in solution interacts and connects, easily forming a mesh structure, making the overall mesh conformation more stable and macroscopically exhibiting good resistance to temperature and salt.
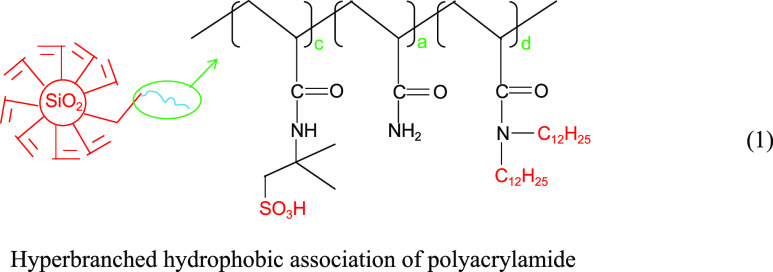
1

**Figure 2:**
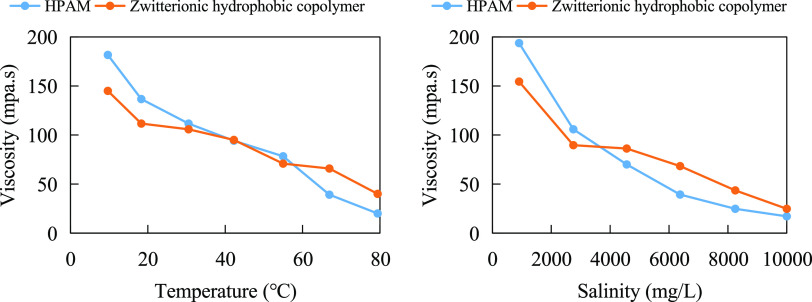
Determination of temperature and salt resistance of HPAM and the amphoteric hydrophobic copolymer (rotor: S62; rotation speed: 30 RPM).

Zhang et al.^13^ synthesized an amphiphilic hydrophobic copolymer, which is shown in the structural Formula 2. As shown in Figure 2, the viscosity of both amphiphilic hydrophobic copolymers and HPAM showed a decreasing trend with the increase of temperature and mineralization, but the decrease of the former was smaller. The viscosity of HPAM is slightly higher than that of the amphoteric hydrophobic copolymer under low-temperature and low-mineralization conditions. The viscosity of amphoteric hydrophobic copolymer is higher than that of HPAM under high-temperature and high-salt conditions. In aqueous solutions, amphoteric hydrophobic copolymers exhibit low viscosity due to the attraction of anionic and cationic groups and the contraction of molecular chains. When the temperature and mineralization increase, on the one hand, the amphoteric hydrophobic copolymer has enhanced heat-absorbing associations due to the presence of more hydrophobic groups; on the other hand, the electrical properties of the anionic and cationic groups are shielded by the electrolyte in the highly mineralized solution, the gravitational interaction between the groups is reduced, and the molecular ductility is enhanced. Therefore, it shows good overall resistance to temperature and salt.
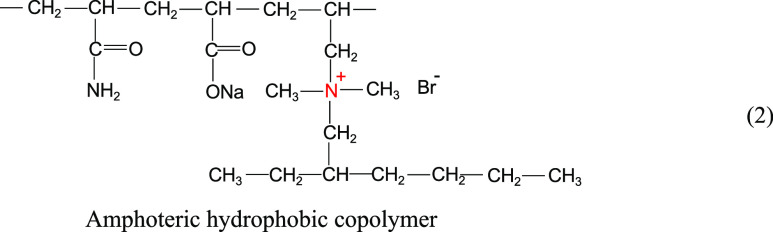
2

**Figure 3:**
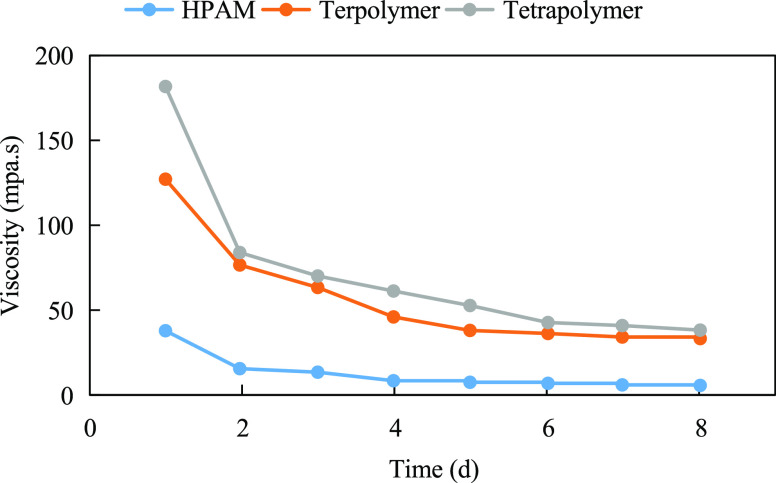
Variation of viscosity with time for different polymer solutions under high-temperature and high-salt conditions (rotor: S62; rotation speed: 30 RPM).

Zhang et al.^14^ also synthesized terpolymers and tetrapolymers containing cyclic structures and sulfonic acid groups, as shown in structural Formulae 3 to 4. As shown in Figure 3, the solutions of HPAM, terpolymer, and quaternary copolymer with the same concentration showed a decreasing viscosity with time under the conditions of 80 °C and mineralization of 10,000 mg/L. However, the viscosity–retention–size relationship of the three is always tetramer > terpolymer > HPAM. On the one hand, the strong spatial resistance provided by the ring structure helps to stabilize the spatial structure of the molecule itself and attenuate the degradation of the molecular chain at high temperatures. On the other hand, the sulfonic acid group is a strong electrolyte group and has strong hydrogen bonding. The electrostatic repulsion between the groups facilitates the extension of molecular chains, and hydrogen bonding facilitates the enhancement of the bonding strength between molecules and keeps the molecular structure stable. So the quaternary copolymer and terpolymer as a whole show good resistance to temperature and salt.
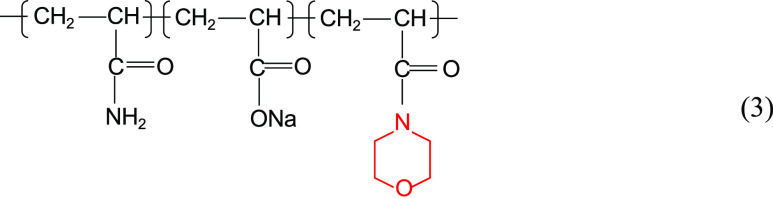
3
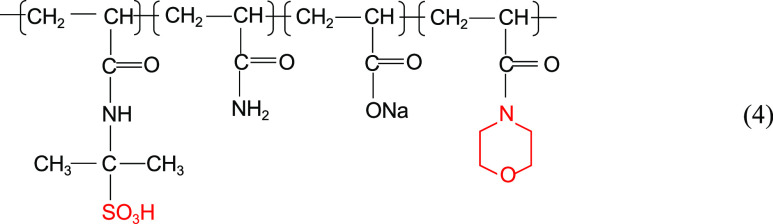
4

**Figure 4:**
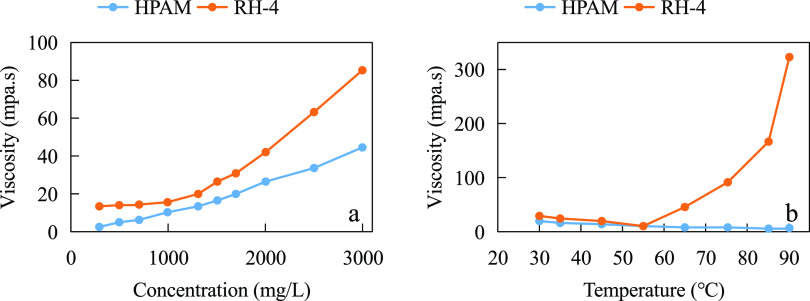
RH-4 temperature and salt resistance test: (a) viscosity–concentration curve and (b) temperature resistance test.

**Figure 5:**
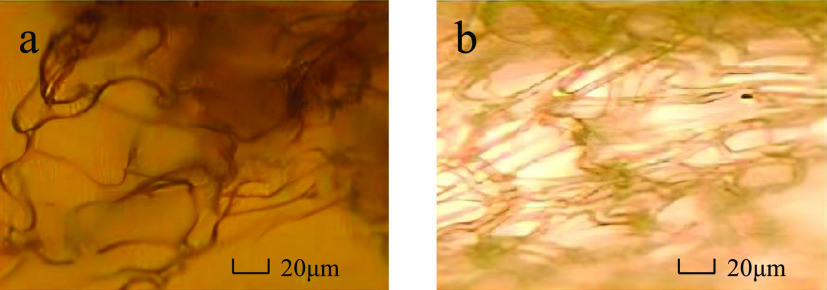
Microscopic morphology of HPAM and RH-4 solutions: (a) HPAM solution and (b) RH-4 solution.

Sarsenbekuly et al.^15^ compared the hydrophobic chain-modified polyacrylamide copolymer RH-4 (see structural Formula 5) with HPAM in the Tahe oil field, Xinjiang, China. As shown in Figure 4, the viscosity of both HPAM and RH-4 solutions increased with increasing concentration under the conditions of 55 °C and mineralization of 9583.74 mg/L (Ca^2+^ + Mg^2+^ ion content of 129 mg/L), and the viscosity of the RH-4 solution was higher. Under the same concentration conditions, the viscosity of HPAM solution decreased with the increase in temperature, while the viscosity of RH-4 solution showed a significant increasing trend with the increase in temperature. With the increase of mineralization, the solution polarity increases, the solubility of hydrophobic chain-modified RH-4 decreases, and the intermolecular hydrophobic chain association increases, which weakens the phenomenon of molecular curling by ion compression diffusion bilayer in solution. Intermolecular hydrophobic chain bonding is a heat-absorbing and entropy-increasing phenomenon that is more likely to occur under high-temperature conditions. As shown in Figure 5, the RH-4 molecule clearly showed a reticulated structure under high-temperature and high-salt conditions, while the HPAM molecule tended to be more linear in structure.
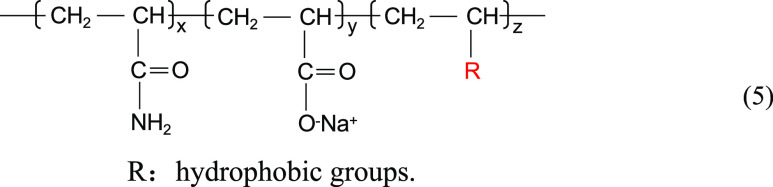
5

#### 2.1.2 Temperature and Salt Resistance Mechanism of Viscosity-Enhancing Copolymers

**Figure 6:**
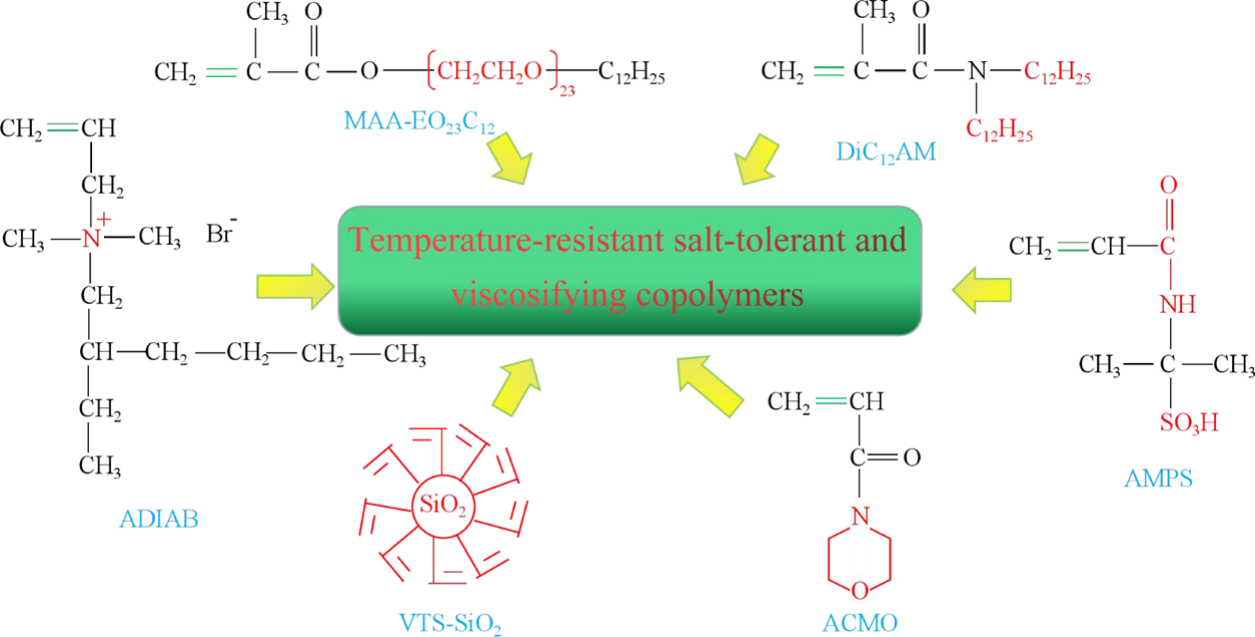
Characteristic functional groups for temperature- and salt-resistant viscosity-building copolymers. AMPS: 2-acrylamido-2-methylpropane sulfonic acid; DiC_12_AM: N,N-di-n-dodecylacrylamide; MAA-EO_23_C_12_: 3, 6, 9, 12, 15, 18, 21, 24, 27, 30, 33, 36, 39, 42, 45, 48, 51, 54, 57, 60, 63, 66, 69-tricosaoxahenoctacontyl methacrylate; VTS-SiO_2_: vinyltriethoxysilane; ADIAB: allyldimethylisooctylammonium bromide; and ACMO: acryloylmorpholine.

Conventional polymer molecules show different degrees of degradation and curling under high temperatures and high-salt conditions, resulting in a significant decrease in the apparent viscosity of their aqueous solutions. The characteristic functional group, on the other hand, due to its unique temperature and salt resistance, enables the copolymer introduced into it to exhibit good viscosity enhancement under high-temperature and high-salt conditions. The common functional groups with temperature and salt resistance properties in copolymers are shown in Figure 6.

**Figure 7:**
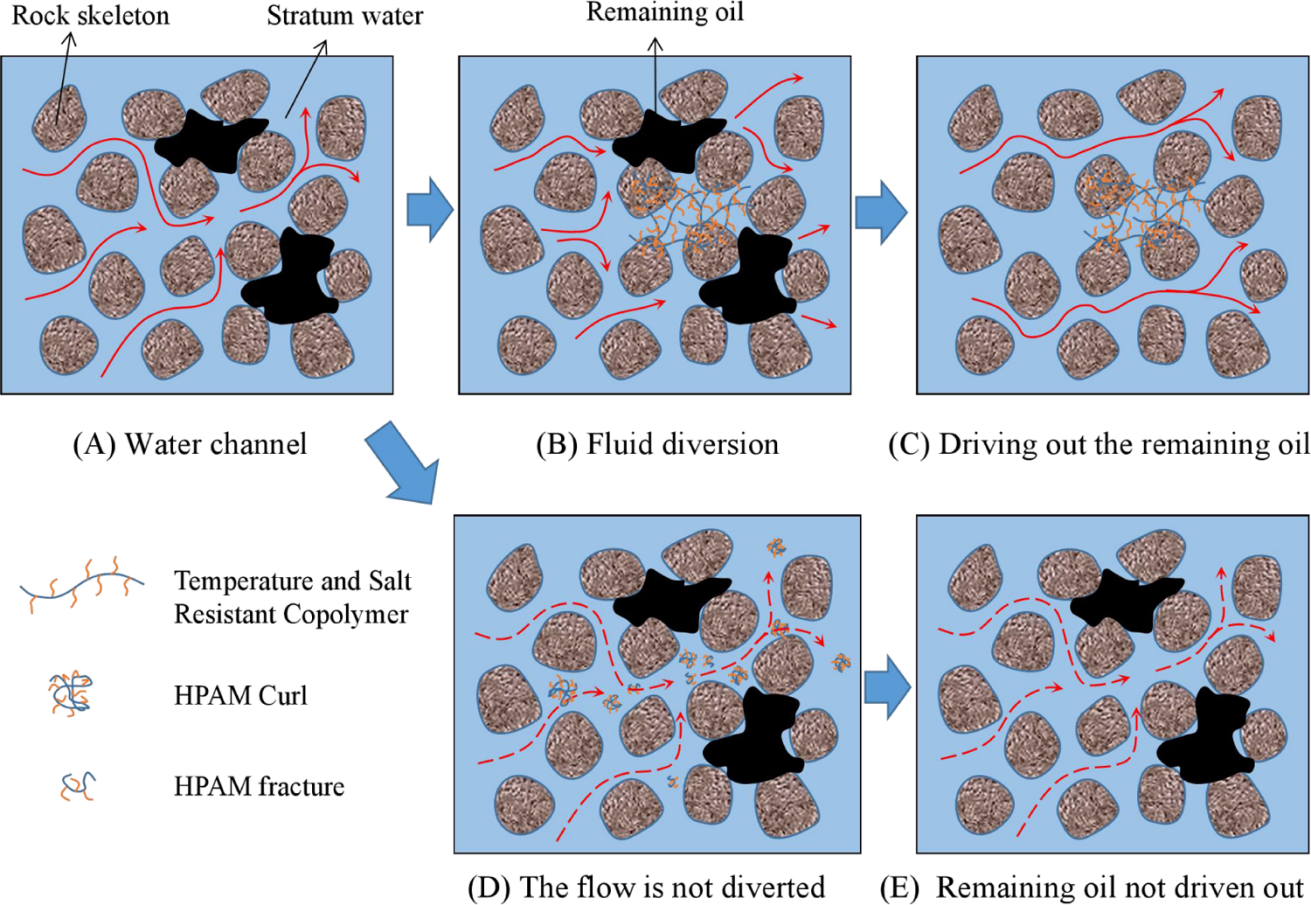
Schematic diagram of the principle of temperature- and salt-resistant copolymers for enhanced recovery.

The temperature and salt resistance mechanisms of the characteristic functional groups in viscosity-enhancing copolymers are as follows. (1) Hydrophobic alkyl chains: In aqueous solutions, hydrophobic alkyl linkage absorbs heat and increases entropy, which is easy to occur under high-temperature conditions. The addition of salt increases the polarity of the solution, makes the nonpolarity of hydrophobic alkyl chains more prominent, decreases the overall solubility, facilitates the association of hydrophobic alkyl chains, promotes intermolecular connections, tends to form a spatial network structure, and increases the apparent viscosity. The hydrophobic alkyl chains are electrically neutral and are less electrostatically shielded by external counterions, enhancing the resistance of the macromolecular chains to electrolytes in solution. The high spatial potential energy of hydrophobic alkyl chains increases their rigidity and improves the strength of the bonded structure.^16^ (2) Nanosilica-centered dendritic structure or other dendritic structures: Compared with linear polymers, the molecular conformation of dendritic structures is more stable, and the dendritic structures are intertwined with each other and easily form a high-strength mesh structure.^17^ Moreover, a certain amount of water molecules are enveloped in it, which helps to keep the polymer molecules in a stable swollen state.^18^ Under the action of external shear, the smaller branched chains reduce the impact on the main chain through their own breakage, which is conducive to improving the shear resistance of copolymers.^19,20^ (3) Ethoxy: Its hydrogen bonding with acrylamide enhances the bonding strength between molecular chains.^21^ The strong hydration ability of ethoxy is conducive to inhibiting the hydrolysis of copolymers. The repulsive forces between the ethoxy and hydrophobic chains facilitate the tendency of the macromolecule to extend its state. The lone pair of electrons of oxygen in ethoxy can occupy the vacant orbitals of high-valent metal ions, forming complexation products and improving the overall hydrophobicity. In addition, with increasing salinity, ethoxy has a tendency to form hydrophobic alkyl chains. (4) Sulfonic acid groups: The strong electrostatic repulsion between the sulfonic acid groups facilitates the enhancement of molecular ductility. The sulfonic acid group is easily hydrogen bonded to the amide group, which facilitates the reduction of amide group degradation.^14^ The sulfonate is weakly attracted to positive ions, is less influenced by counterions, has strong hydration, and is favorable to inhibiting amide group hydrolysis at high temperatures. The closed amide bond in AMPS facilitates the improvement of the overall temperature resistance, and the large side chains facilitate the improvement of the overall spatial site energy, making the polymer molecular chain more rigid, so the overall thermal stability is higher.^22^ (5) Cyclic acryloyl morin: Hydrolysis to cations facilitates increased solubility of copolymers. The ring structure facilitates the increase of spatial potential resistance, enhances the rigidity of the chain, and keeps the chain ductile, thus improving the overall temperature resistance.^14^ (6) Anionic and cationic amphoteric groups: In the case of low salinity, electrostatic attraction causes the molecular chains to curl and the apparent viscosity to be small.^23^ However, as the salt content increases, metal ions shield the electrical properties of the polyelectrolyte, leading to the stretching of molecular chains and an increase in apparent viscosity. The performance of temperature- and salt-resistant copolymers and HPAMs under high-temperature and high-salt reservoir conditions is shown in Figure 7.

### 2.2 Temperature- and Salt-Resistant Low-Interfacial-Tension Surfactant

Surfactants in tertiary oil recovery can reduce oil–water interfacial tension and are indispensable main agents for improving oil drive efficiency.^24,25^ The loss of surfactant adsorption in oil formations and high-temperature and high-salt failure are the two major problems facing oil fields.^26−29^ Under high temperatures and high- salt conditions, the performance of surfactants is reduced in two main ways:^30^ (1) The increase in solution electrolyte concentration severely compresses the double electric layer and reduces the thickness of the oil–water interface layer, forcing the surfactant to dissolve in the oil phase, resulting in an increase in interfacial tension. (2) The temperature increases, the molecular structure is destroyed, and the performance deteriorates, leading to an increase in interfacial tension.

In recent years, many studies have been conducted on low-interfacial-tension surfactants under high-temperature and high-salt conditions, and there are many compound surfactants that achieve ultralow interfacial tension under high-temperature and high-salt conditions,^31,32^ as well as single surfactants and surfactant systems that do not achieve ultralow interfacial tension under high-temperature and high-salt conditions but can maintain low interfacial tension for a long time.^33−36^ Surfactants containing fluorine and silicon have better surface properties under high temperatures and high-salt conditions, but they are mostly limited to indoor studies due to their high cost.^37^ As the research progresses, it is expected to be applied to enhanced oil recovery technology on a large scale.

#### 2.2.1 Synthesis and Properties of Low-Interfacial-Tension Surfactants

**Figure 8:**
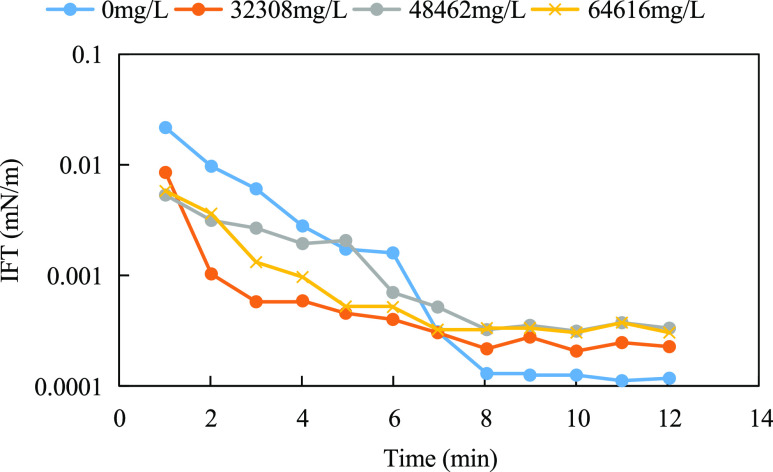
Temperature and salt resistance test of a nonylphenol betaine amphoteric surfactant.

Ding et al.^38^ synthesized a nonylphenol betaine amphoteric surfactant with the structural formula shown in (6). As shown in Figure 8, the interfacial tension between the surfactant solution and the crude oil was stable on the order of 10^–4^ mN/m for the interval of 85 °C and mineralization of 0–64,616 mg/L. In the molecular structure, the benzene ring is not charged and is less affected by the electrolyte of the solution, which improves the stability of the molecular structure. The anionic group is chelated with the metal ions in the solution, which facilitates the enhancement of the stability of the molecular structure. The repulsive effect of cations on metal ions in solution facilitates the weakening of electrolyte effects on molecular head groups. The good hydration properties of the sulfonic acid group facilitate the reduction of the salting effect under high mineralization conditions. C–S with high bonding energy facilitates the reduction of high-temperature degradation of molecules. Therefore, the overall performance has good resistance to temperature and salt.
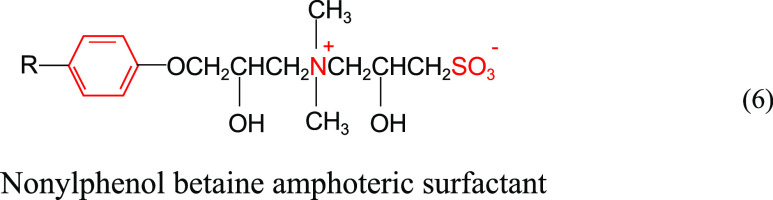
6

**Figure 9:**
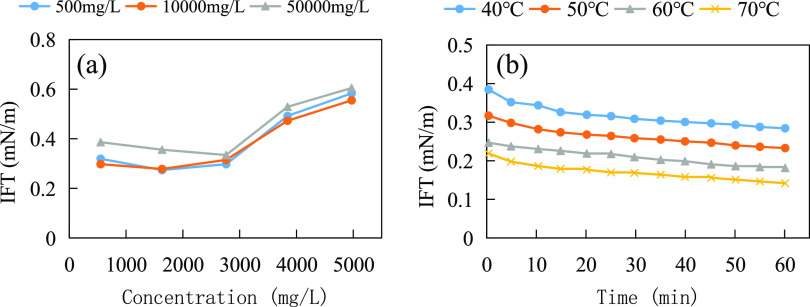
Temperature and salt resistance test of ammonium nonylphenol ethoxylate sulfate: (a) salt resistance test and (b) temperature resistance test.

Mao^39^ synthesized nonylphenol polyoxyethylene ether ammonium sulfate with the structural formula shown in (7). As shown in Figure 9a, the differences in interfacial tension between nonylphenol polyoxyethylene ether ammonium sulfate solution and crude oil were not significant for 500 and 10,000 mg/L mineralization conditions. When the mineralization was increased to 50,000 mg/L (Ca^2+^ + Mg^2+^ ion content of 830 mg/L), the rise in interfacial tension was not high, reflecting the good salt resistance performance. As shown in Figure 9b, the interfacial tension tends to decrease with increasing temperature, reflecting good temperature resistance. In the molecular structure, ethoxy is not charged and is little affected by the electrolyte in solution, and the hydrogen bonding with water molecules facilitates the improvement of the overall water solubility of the molecule. The strong hydration properties of the sulfonic acid group and the stabilizing effect of the benzene ring on the molecular structure attenuate the high-temperature degradation of the molecule. The molecule as a whole showed good resistance to temperature and salt.
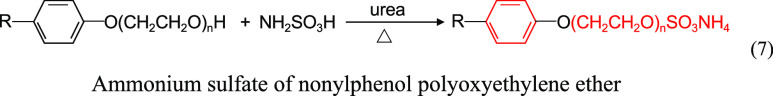
7

**Figure 10:**
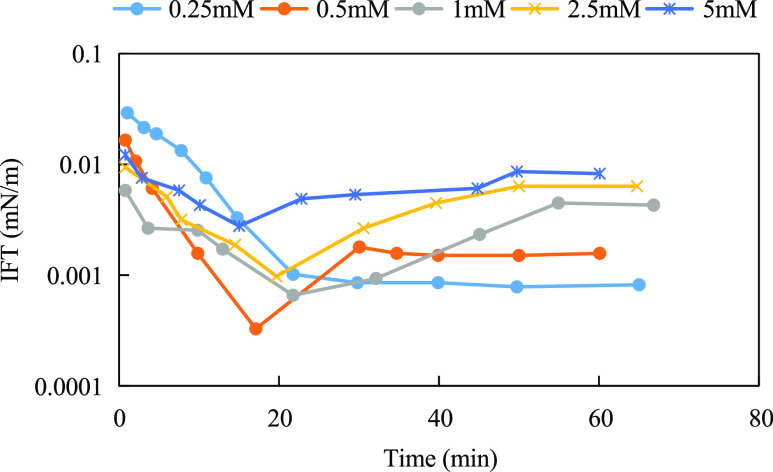
Dynamic interfacial tension of solutions of binary mixtures with different molar concentrations.

Li et al.^40^ synthesized surfactants of 1,3-dialkyl glycerol ether derivatives with the structural formulae shown in (8) and (9). The two series were compounded into binary mixtures according to the molar fraction 4:1, and the interfacial tension between the solutions of different concentrations of binary mixtures and crude oil was tested at 60 °C with a total mineralization of 20,000 mg/L (Ca^2+^ + Mg^2+^ ion content of 840 mg/L). As shown in Figure 10, the interfacial tension of the solutions of binary mixtures with different concentrations can be stabilized in the range of 10^–3^ to 10^–4^ mN/m. In the molecular structure, the double hydrocarbon chain facilitates the increase of nonpolar group density at the oil–water interface and enhances the surface chemical properties of the molecule. The higher C–C bonding facilitates the stabilization of the molecular structure and reduces thermal degradation. The anionic and cationic groups, ethoxy and sulfonic acid groups, not only have the function of stabilizing the molecular structure but also improve the overall water solubility. The synergistic effect of multiple characteristic functional groups enables the molecule as a whole to exhibit good resistance to temperature and salt.
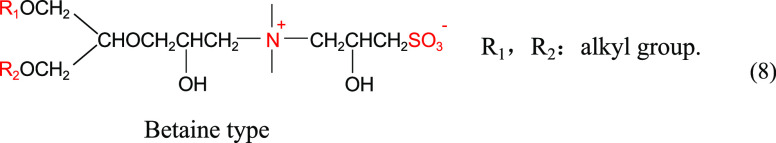
8
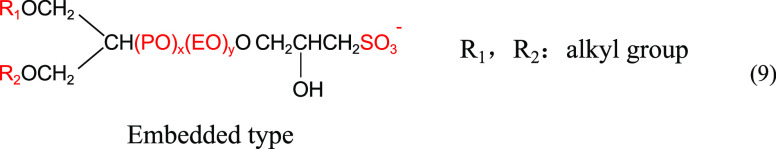
9

**Figure 11:**
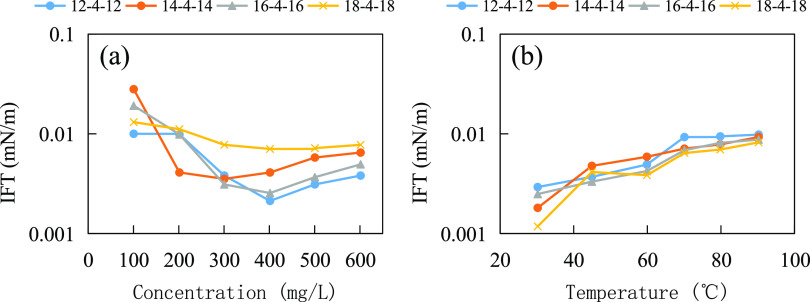
Temperature and salt resistance tests of amphoteric biosurfactants: (a) salt resistance test and (b) temperature resistance test.

A serial amphoteric gemini surfactant was synthesized by Ren et al.^41^ The structural formula is shown in (10). Surfactants with single-chain carbon contents of 12, 14, 16, and 18 were tested for temperature and salt resistance. As shown in Figure 11a, under the condition of 40,530 mg/L mineralization (Mg^2+^ ion content of 153 mg/L), the interfacial tension between all four surfactant solutions and crude oil decreased slightly with the increase in surfactant concentration, and the values were basically on the order of 10^–3^ mN/m, reflecting the good salt resistance performance. As shown in Figure 11b, the interfacial tension between the four surfactant solutions and crude oil increased slightly with the increase in temperature, and the values were basically on the order of 10^–3^ mN/m, reflecting good temperature resistance. In the molecular structure, the double cationic and double sulfonic acid groups facilitate the strengthening of the group’s anti-temperature and anti-salt effects. The synergistic effect of multiple characteristic functional groups enables the molecule as a whole to exhibit good resistance to temperature and salt.
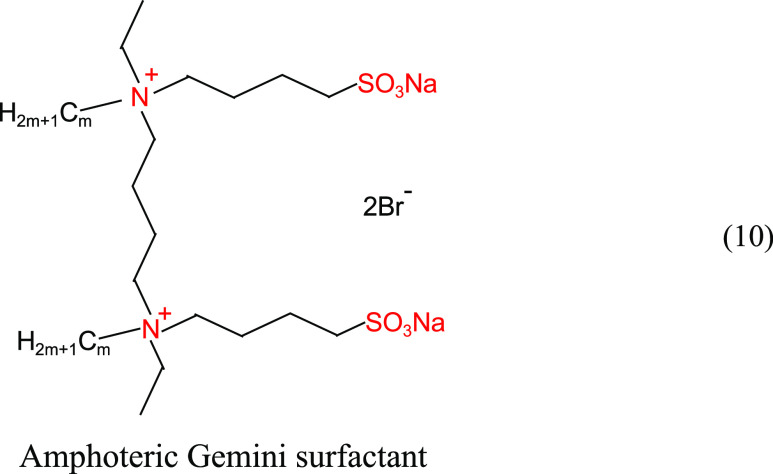
10

**Figure 12:**
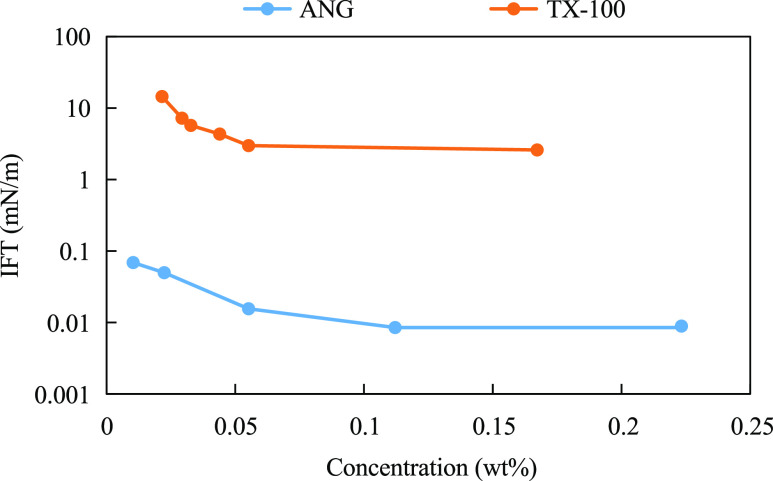
Temperature and salt resistance test of ANG Gemini surfactant.

Hou et al.^42^ synthesized a gemini surfactant, ANG, with the structural formula shown in (11). As shown in Figure 12, the interfacial tension between ANG and conventional TX-100 solutions and crude oil showed a decreasing trend with increasing surfactant concentration at 120 °C and a total mineralization of 188,870 mg/L (Ca^2+^ + Mg^2+^ ion content of about 900 mg/L). However, the interfacial tension of ANG is on the order of 10^–2^–10^–3^ mN/m, and the performance of reducing interfacial tension under high-temperature and high-salt conditions is significantly better than that of TX-100. The double benzene ring, double sulfonated groups, and several ethoxy groups in the molecular structure help to stabilize the molecular structure and improve the overall water solubility. ANG surfactants exhibit good resistance to temperature and salt.
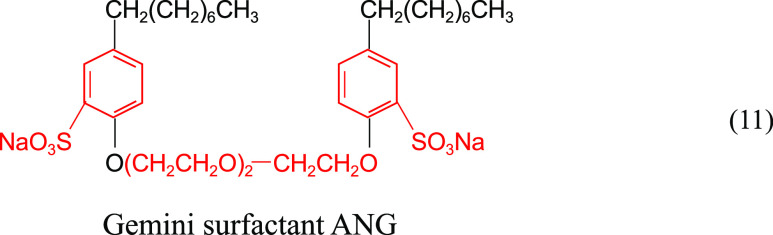
11

**Figure 13:**
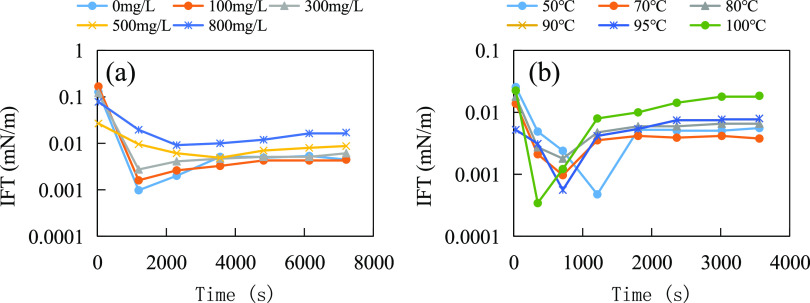
Temperature and salt resistance test of SPOPD bio-based amphoteric surfactant: (a) salt resistance test (interfacial tension at different Ca^2+^ ion concentrations) and (b) temperature resistance test.

Chen et al.^43^ synthesized a bio-based amphoteric surfactant, SPOPD, with the structural formula shown in (12). As shown in Figure 13a, the interfacial tension between the 0.5 g/L SPOPD solution and crude oil tends to increase slightly with increasing Ca^2+^ ion concentration. When the Ca^2+^ ion concentration is below 500 mg/L, the equilibrium interfacial tension is of the order of 10^–3^ mN/m. When the Ca^2+^ ion concentration reaches 800 mg/L, the equilibrium interfacial tension is of the order of 10^–2^ mN/m, which is still in the low interfacial tension range. The interfacial tension between the 0.5 g/L SPOPD solution and crude oil decreased and then increased as the temperature increased. As shown in Figure 13b, the equilibrium interfacial tension is of the order of 10^–3^ mN/m when the temperature is below 95 °C. When the temperature reaches 100 °C, the equilibrium interfacial tension is of the order of 10^–2^ mN/m, which is still in the low interfacial tension range. The benzene ring and amphoteric groups in the molecular structure facilitate the stabilization of the molecular structure and improve the overall water solubility. SPOPD surfactants exhibit good resistance to temperature and salt.
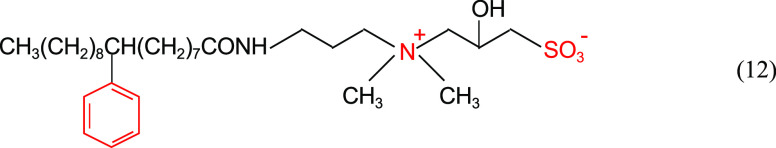
12

#### 2.2.2 Temperature and Salt Resistance Mechanisms of Low-Interfacial-Tension Surfactants

**Figure 14:**
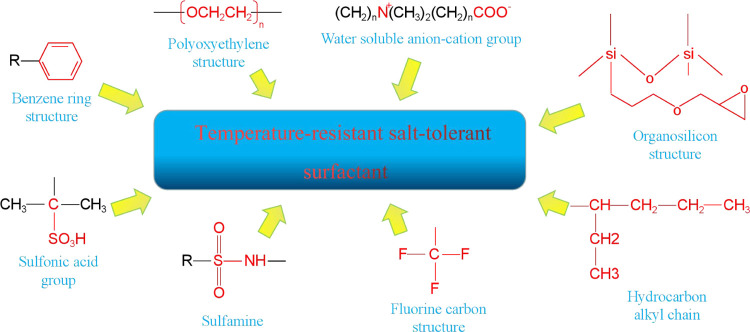
Relationship between characteristic functional groups and temperature and salt-resistant low-interfacial-tension-surfactants.

The above new surfactant molecules contain two or more functional groups such as ethoxy groups, sulfonic acid groups, benzene rings, anionic and cationic groups, and double hydrocarbon chain structures. Sulfonamide, fluorocarbon, and silicone structures have also been used in the design of temperature- and salt-resistant surfactant molecular structures. These characteristic functional groups give the surfactant good low interfacial tension properties under high-temperature and high-salt conditions (see Figure 14).

**Figure 15:**
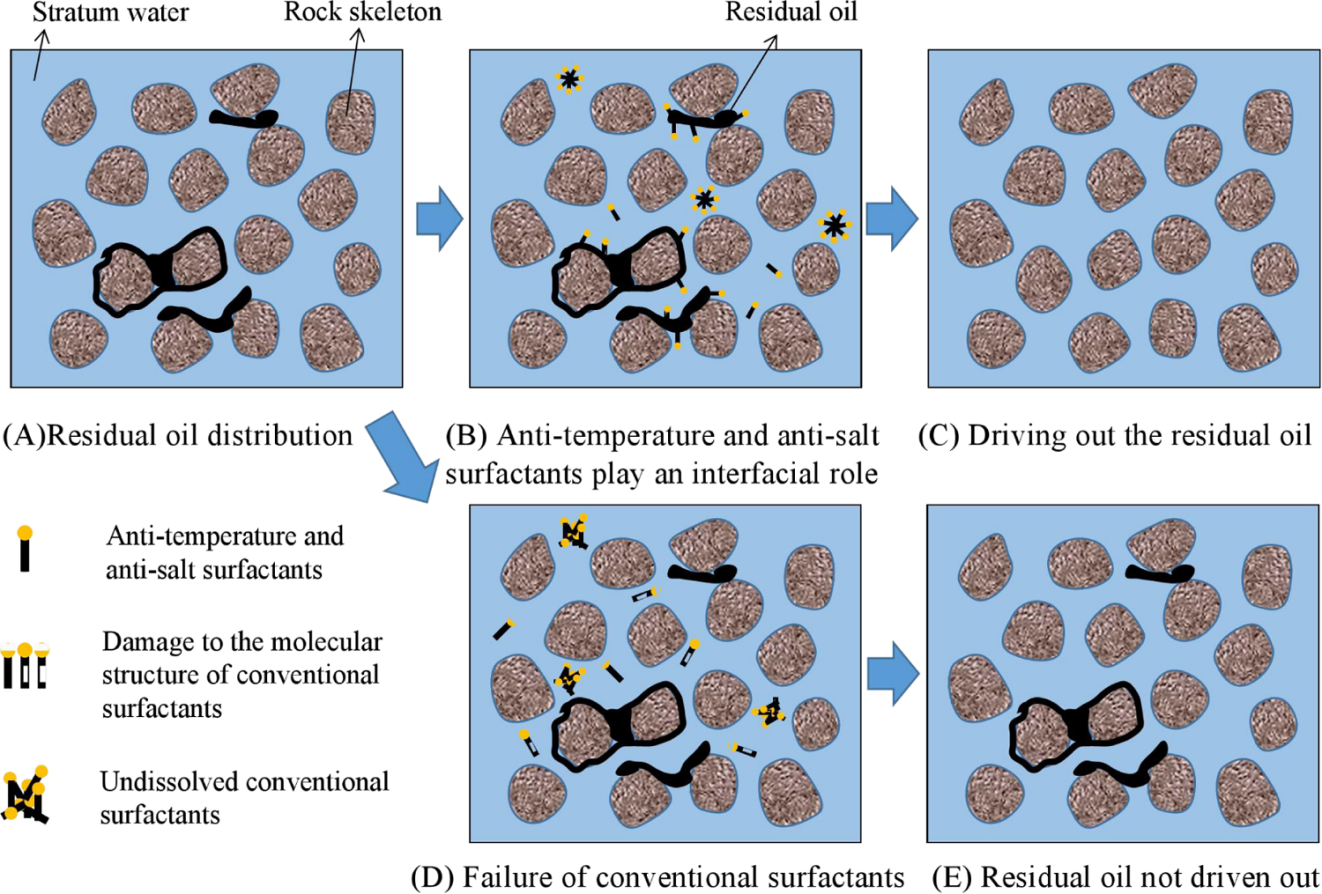
Schematic diagram of the principle of enhanced recovery with anti-temperature and anti-salt surfactants.

The mechanism of temperature and salt resistance of the characteristic functional groups in low-interfacial-tension surfactants is as follows. (1) Ethoxy: Ethoxy is weakly hydrophilic and is not affected by electrolytes. It has strong hydrogen bonding with water molecules and is attached to strong hydrophobic chains to improve overall water solubility. To a certain extent, the increase in the number of ethoxy groups makes the surfactant shift from a strongly nonpolar to a weakly hydrophobic to a weakly hydrophilic gradient, which is conducive to making the surfactant monomolecular layer have strong and equal interactions with both oil and water phases, making it easy to achieve low oil–water interfacial tension performance.^33,34^ (2) Double hydrocarbon chain: Double hydrocarbon chain surfactants have a higher hydrocarbon density at the oil–water interface, and their hydrophobicity is better than that of single hydrocarbon chain surfactants, which can easily improve the differentiation of amphiphilic structures.^40^ The high C–C bond energy in the double hydrocarbon chain makes it less susceptible to degradation at high temperatures and less affected by electrolytes. (3) Benzene ring: The benzene ring is not charged and can improve molecular rigidity, which helps stabilize the overall structure of the molecule and reduce the effect of high-temperature degradation and mineralization.^38,39^ (4) Amphoteric structure: Amphoteric surfactants have a chelating effect on metal ions, which facilitates the enhancement of overall molecular stability. The positive charge in the amphoteric surfactant has a repulsive effect on the metal ions in the solution, which facilitates the weakening of the damage to the hydration film of the surfactant head base by excess salt.^38,40,43^ (5) Sulfonic acid group: The sulfonic acid group has good hydration properties as a hydrophilic group, and the anti-salting effect is obvious. The high C–S bond energy at the connection site facilitates the improvement of the overall molecular thermal stability. (6) Fluorocarbon structure: F has a strong negative charge, a high oxidation potential, and a high ionization energy, which makes the F–C bonding energy strong. The size of F atoms is moderate, just enough to shield and protect the C–C bond, which is conducive to the stability of the overall molecular structure. The low polarization of F and the low polarity of the F–C bond lead to a strong hydrophobic interaction of the molecule and low phase repulsion, which facilitates aggregation on the surface. (7) Sulfonamide structure: The sulfonamide structure is relatively stable, and the shielded sulfonamide in the molecule can make the sulfonic acid group have good resistance to divalent cations so that the overall molecule has good hydrolytic stability, which is conducive to improving acid and alkali resistance and high-temperature resistance. (8) Organic silicon: The Si–O bond in the silicone structure has low rotational energy and low cohesion energy and is highly flexible, which facilitates the solubility of molecules in water, organic solvents, and even supercritical CO_2_, thereby reducing the surface tension of these media to very low levels. The performance of anti-temperature and anti-salt surfactants and conventional surfactants under high- temperature and high-salt reservoir conditions is shown in Figure 15.

### 2.3 Commonality of Molecular Structures of Temperature- and Salt-Resistant Viscosity-Enhancing Copolymers and Low-Interfacial-Tension Surfactants

**Figure 16:**
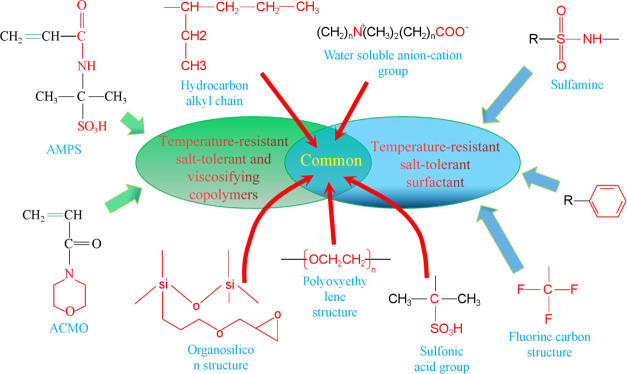
Relationship between characteristic functional groups and temperature- and salt-resistant oil displacement agents.

Comparing the synthesis of anti-temperature and anti-salt type chemicals for displacing oil in recent years, it was found that there are some commonalities in the molecular structures of viscosity-enhancing copolymers and low-interfacial-tension surfactants (see Figure 16). Both types of substances can introduce ethoxy groups, sulfonation groups, water-soluble anionic and cationic groups, alkyl chains, silicone (oxygen) alkyl groups, and other characteristic functional groups to make the aqueous solution of chemical agents excellent in temperature and salt resistance.

The common structure of the two types of substances has obvious effects on temperature and salt resistance, with similar and different mechanisms of action. For viscosity-enhancing copolymers, alkyl chains mainly play the role of hydrophobic linkage; water-soluble anionic and cationic groups mainly reflect the shielding of metal ions in solution; ethoxy groups mainly reflect hydrogen bonding and repulsion with hydrocarbon chains; sulfonated groups mainly reflect hydrogen bonding and strong hydration to inhibit the degradation and hydrolysis of amide groups; and silicon mainly branched the molecule and enhanced the strength of the spatial network structure. For surfactants, alkyl chains are mainly reflected in improving the differentiation of hydrophilic and lipophilic groups; water-soluble anionic and cationic groups are mainly reflected in chelating with metal ions to enhance stability; ethoxy groups are mainly reflected in regulating nonpolar strength and improving solubility to a certain extent; sulfonated groups are mainly reflected in good hydration performance and anti-salting effect; and silicon is mainly reflected in the flexibility of the Si–O bond and thus improving solubility.

## 3 Conclusions

The development of temperature- and salt-resistant viscosity-enhancing copolymers and low-interfacial-tension surfactants is a trend in the development of oil field chemicals, and the introduction of characteristic functional groups is the key to improving the temperature- and salt-resistant performance. Temperature- and salt-resistant viscosity-enhancing copolymers and low-interfacial-tension surfactant molecules have certain commonalities in terms of temperature- and salt-resistant property functional groups. The different properties of the two types of substances inevitably lead to differences in the efficiency of the introduction and the performance of the same characteristic functional group under the same conditions. The commonality in molecular structure provides a reference for the design and synthesis of novel temperature- and salt-resistant chemical agent molecules. The molecular structure can be designed by the arrangement and combination of functional groups with temperature and salt resistance properties to obtain high-performance target products. New temperature- and salt-resistant functional groups are to be studied, discovered, and verified, and the temperature- and salt-resistant performance of oil displacement agents can be further improved by improving the molecular structure.
